# Barbary macaques show sex-related differences in body weight based on anthropogenic food exposure despite comparable female–male stable isotope ratios

**DOI:** 10.1038/s41598-024-53641-9

**Published:** 2024-02-09

**Authors:** Sana T. Saiyed, Agustin Fuentes, Eric Shaw, Mark R. Schurr, Lee T. Gettler

**Affiliations:** 1 Department of Anthropology, University of Notre Dame, Notre Dame, IN USA; 2 Department of Anthropology, Princeton University, Princeton, NJ USA; 3 Gibraltar Ornithological and Natural History Society (GONHS), Gibraltar, Gibraltar

**Keywords:** Ecology, Stable isotope analysis, Biological anthropology

## Abstract

As the human–primate interface expands, many nonhuman primate (NHP) populations exploit anthropogenic foods to survive, while some populations opportunistically target them. Though anthropogenic food consumption is sometimes associated with greater reproductive output and survival in these populations, there is a dearth of research on possible health effects. We explore how differential exposure to anthropogenic foods is linked to variation in isotopic compositions (δ^13^C and δ^15^N) and body weights in Barbary macaques (*Macaca sylvanus*) in the Upper Rock Nature Reserve, Gibraltar. We placed monkeys into three categories based on anthropogenic food exposure. We then analyzed individuals for isotopic signatures (N = 147) and body weight measurements (N = 80). Using the lowest exposure category as the comparison, we found body weights and δ^15^N values, but not δ^13^C values, significantly differed across key categories. Within categories, we found no significant associations between sex and δ^13^C or δ^15^N values, suggesting that individuals within categories consumed similar foods regardless of sex. We found a significant interaction effect between category and sex for predicting body weights. These results suggest that sex plays a role in how anthropogenic foods are accessed and consumed regardless of exposure, which may result in differential health profiles for female and male macaques.

## Introduction

Humans contribute to immense changes in non-human species’ ecosystems through various processes such as agriculture, deforestation, and urbanization. As a result, animal populations are forced to adjust their patterns of behavior to respond. One such adjustment is in diet and foraging, often due to the decrease of a population’s natural food source^1–3^. These populations may shift away from consuming natural resources in favor of anthropogenic foods because of the increased abundance and accessibility of these resources compared to their natural foods as a result of human-imposed changes to local ecologies. Studies demonstrate that wildlife in urban and semi-urban areas are supplementing their diets with anthropogenic food sources and populations that are able to do so successfully tend to thrive, in terms of fitness-relevant indicators, with longer breeding seasons and shorter interbirth intervals, larger troop sizes, and overall population increases^4,5^. However, because animals’ physiological and behavioral profiles are often adapted to specific diets and/or relatively constrained resource availability, these adjustments in food consumption have the potential to negatively impact their health.

Anthropogenic foods that are cooked or otherwise highly processed are differentially digested than the naturally occurring raw foods that wildlife typically rely on^6^, and non-human populations living in urban environments are often consuming anthropogenic foods that are calorically dense, including with high fat and sugar content^4^. Macaque monkeys (genus *Macaca*)—the primate of focus for the present analyses—are a taxonomic group that is successful at thriving in human-constructed spaces, primarily by diversifying its diet to include human foods. Macaques living near human populations, or commensal macaques, actively search for and consume human foods to supplement their naturally foraged diet of vegetation and, to a lesser degree, fruits and insects^7,8^. There is currently a dearth of research on how human foods and their nutritional content are linked to macaque health, with both positive and negative effects being possible. For example, research demonstrates that the consumption of anthropogenic food can buffer macaque populations during seasons when their nutritionally-rich natural foods may be low^9^. However, other work shows that consuming discarded anthropogenic foods can increase rates of bacterial parasite presence in commensal macaque populations^8^. This is an especially timely area of research because as human populations continue to increase and expand, nonhuman primates (NHPs) are forced to exploit urban areas for food in order to survive or they risk perishing^10,11^. For reasons described above, macaques are a well-suited model genus for studying the possible relationships between anthropogenic foods and health given their demonstrated preference for anthropogenic foods over natural foods and ability to succeed in urban areas across the globe^4,10,11^.

### Macaques and anthropogenic food

Macaques’ natural (non-anthropogenic) diets generally consist of fruits, leaves, and vegetation that are rich in fiber and low in fats and sugars^12,13^. Across much of Asia and in Gibraltar and North Africa, macaques are fed and also forage for high-calorie anthropogenic foods. In many cases, anthropogenic foods are highly preferred and experimental lab studies have shown evidence of increased adiposity in NHPs consuming a similar high-calorie diet^14,15^. Though the consumption of these foods has been linked to increased reproductive output and overall survival rates in wild NHP populations, little research has investigated whether it also leads to possible phenotypic profiles that could have adverse health effects, such as atypical weight gain and fat deposition in wild populations^16^.

Access to and consumption of these foods is known to depend on factors such as sex and rank across NHPs. In many species, NHP males tend to have priority access to highly desired foods, which include tourist offerings and anthropogenic foods in general, due to dominance hierarchies, risky behaviors, and aggression around food^17,18^. Research on macaques finds that adult males are more likely than females to interact with humans for food and, as a result, incorporate anthropogenic foods into their diets more^19,20^. Marty and colleagues observed troops from three different macaque species (rhesus, long-tailed, and bonnet) that had high exposure to anthropogenic food resources through tourist and local feeding. They found that male diets reflected higher proportions of anthropogenic food intake than the diets of females within the same troop^4^. Studies of wild populations anecdotally report that NHP individuals that consume more anthropogenic foods typically exhibit weight gain, so one could predict that male macaques would show relative increases in body weight compared to females in populations with routine access to anthropogenic foods. However, Marechal et al. found that female macaques showed greater changes in body size and condition than male macaques when consuming anthropogenic foods^21^. Studies of captive populations also suggest a more pronounced impact of high-calorie, anthropogenic foods on female NHPs^22,23^, but few studies have analyzed this in populations in which access to anthropogenic foods differs based on sex^11^.

### Stable isotopes

A first step to investigating possible health impacts of anthropogenic foods on commensal macaques through body weights is to quantify a target population’s consumption of anthropogenic foods by studying their foraging and dietary ecology. Behavioral studies show access to human foods has shifted macaque populations’ foraging ecologies towards preferential consumption of those resources, such as changing activity patterns in hopes of receiving food from tourists^18^, to the extent that human foods may comprise the largest portion of their diet^3,7,20,24^. In addition to behavioral monitoring, stable isotope analyses are useful for investigations into foraging ecology because after an animal consumes foods, the isotopic compositions of those items are then reflected in the animal’s tissues^25^. The most commonly analyzed stable isotopes for dietary investigations are carbon (δ^13^C) and nitrogen (δ^15^N). Because δ^13^C values differ based on the consumption of C3 versus C4 plants, they are particularly useful for exploring levels of anthropogenic food consumption, as processed human foods typically utilize C4 plant ingredients^26–28^. Similarly, δ^15^N values can reflect anthropogenic food consumption because agricultural crops utilize Nitrogen-rich fertilizers, which can then contribute to increased Nitrogen isotopic composition for animals^27^.

Stable isotope analyses are particularly useful in animal dietary studies because they can provide long-term dietary information, including seasonal variation, when time is limited for behavioral monitoring. In general, stable isotope analyses have been employed in studies of NHPs for over 30 years in attempts to understand aspects of diet that other methods, like behavioral observation, would struggle to detect^29,30^. For example, Loudon et al. conducted a study on the feeding ecology of South African vervet monkeys using both behavioral and stable isotope analyses. From behavioral observations, several troops were assumed to have only mid-level access to human foods. However, their stable isotope values suggested higher consumption of human foods, prompting additional behavioral observations that showed that these troops were crop foraging and swimming across rivers without the knowledge of local human populations^26^. Multiple other studies show that isotopic analyses can help detect dietary differences amongst populations or troops^19,31,32^ or be used to compare a population’s diet across time^33^. Because behavioral observations of foraging ecology can sometimes be limited by observer time and wildlife visibility, stable isotope analyses can expand our understandings of diet by providing additional measurements for assessments of diet^30^. To get the most accurate and long-term understanding of human food consumption in macaque populations for health profiles, incorporating stable isotope analyses into dietary investigations is useful.

In this study, we investigated dietary differences and body sizes of Barbary macaques (*M. sylvanus*) from the Upper Rock Nature Reserve (URNR), Gibraltar, using stable isotope and weight data. Troops within the Reserve experience varying levels of anthropogenic interaction and access to food. In a previous study, Schurr and colleagues compared behavioral observations of foraging ecology and δ^13^C and δ^15^N values of five troops in URNR. They found significant differences in both δ^13^C and δ^15^N stable isotope values amongst troops related to their interaction patterns with humans, suggesting various consumption of human foods^19^; they did not examine body weight. In this study, we analyzed hair-based isotope data from 147 individuals (compared to 107 in the prior study^19^) and weight measurements from 80 individuals. As part of this overall increase in sample size, the current analysis adds individuals from a troop that resides in and around a garbage dump, which was not included in the prior study^19^. This additional troop has limited interaction with humans, yet feeds primarily on discarded human foods.

We compared δ^13^C and δ^15^N values and weight measurements between these six troops categorized into three ecological niches reflecting varying exposures to human foods based on the previous study’s observational data (anthropogenic food exposure: Low, Medium, and High; Table 1). We predict that monkeys with greater access to anthropogenic foods will show δ^13^C and δ^15^N profiles associated with human foods (increased δ^13^C values and decreased δ^15^N values) compared to monkeys with less access, which aligns with the previous findings of Schurr et al.^19^. We also predict that monkeys with greater access to anthropogenic foods will show increased body weights than monkeys with the lowest anthropogenic food access. Finally, we predict that associations between anthropogenic food exposure and body weight and δ^13^C and δ^15^N values will be stronger for males than females due to sex-related differences in food access (i.e., moderation by sex).

**Table 1:** Each troop’s level of interaction with tourists and access to anthropogenic foods.

Group name	Tourist interaction	Provisioned by GONHS?	Human foods?	Category
Ape’s Den	Yes	Yes	Yes—often	High
Prince Phillip’s Arch	Yes	Yes	Yes—often	High
Farrington’s Barracks	Sometimes	Yes	Yes—sometimes	Middle
Royal Anglian way	Sometimes	Yes	Yes—sometimes	Middle
Middle Hill	No	Yes	No	Low
Incinerator	No	No	Yes—often; food waste	High

Provisioned foods are non-processed foods, such as fruits, vegetables, and a ‘monkey chow’, that are provided by GONHS. Anthropogenic foods are typically highly processed and high-calorie.

## Methods

### Study species and area

**Figure 1:**
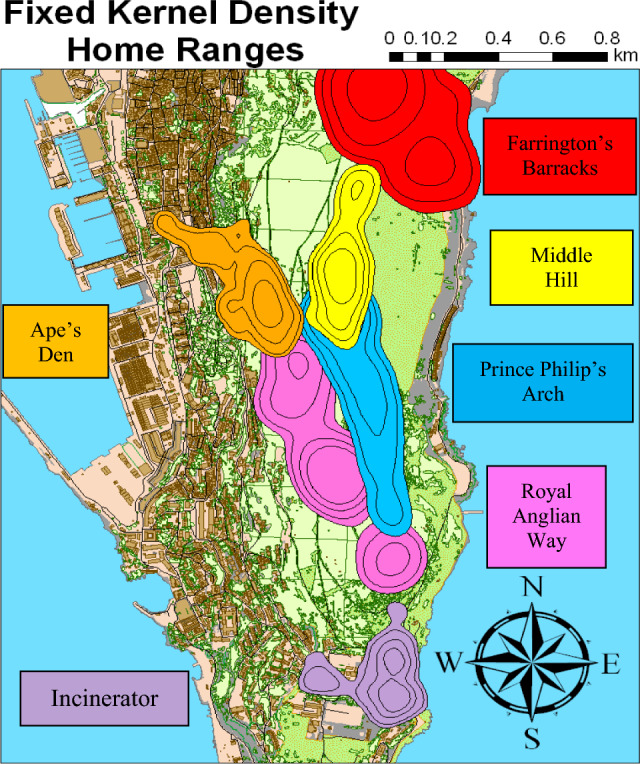
Map indicating range differences amongst the 6 different troops. Colors represent different troops, labeled in nearby boxes added in Microsoft Word, version 16.81: https://www.microsoft.com/en-us/microsoft-365/word. Map created using adehabitat package in R Studio, version 4.3.1: https://docs.posit.co/previous-versions/rstudio/.

The Upper Rock Nature Reserve in Gibraltar was designated in 1993 and is home to over 230 Barbary macaques. The focal macaques of this study comprise 6 distinct troops residing across the 97 ha of the Reserve (Fig. 1): Ape’s Den, Prince Phillip’s Arch, Farrington’s Barracks, Royal Anglian Way, Middle Hill, and Incinerator.

During the period these data were collected, all troops except one were provisioned with non-processed foods such as fruit, vegetables, and a wheat and seed mix twice daily by the Gibraltar Ornithological and Natural History Society (GONHS), a non-governmental organization in Gibraltar. This provisioning initially began in 1918 to minimize food seeking in urban areas. Some troops are also exposed to anthropogenic foods through tourist feedings, which mostly include, but are not limited to, snack food items such as chips. Troops that reside closer to tourist roads and stations are frequently visited and fed an assortment of processed foods by tourists, while other troops reside in areas off-limits to tourists and may not have access to these anthropogenic foods through tourist feedings^19^. The Incinerator troop was the only troop assessed that was not provisioned by GONHS. It also has almost no contact with tourists, but because this troop lives in and around a local incinerator, these individuals frequently consume food waste from people’s homes. The Incinerator troop is thus exposed to high amounts of anthropogenic foods that are highly processed and disposed of into the local incinerator. For the present analyses, we categorized troops into three categories based on exposure to anthropogenic foods (Table 1): Low, Middle, and High. Exposure was determined from previous analyses of foraging behaviors and stable isotopes^19,34^ and, in the case of the Incinerator troop, the authors’ unpublished observations at URNR. For example, because the Middle Hill troop had no documented or observed access to human foods and its reported stable isotope signatures reflected low consumption of anthropogenic foods (low δ^13^C and high δ^15^N;^19,34^), Middle Hill macaques were placed into the “Low” category of anthropogenic food exposure. We report key descriptive statistics for the Low, Middle, and High exposure groups, stratified by sex, in Table 2.

**Table 2:** Average weights and isotope measurements for each category, also shown separated by sex.

	Low Mean (SD) (N)	Middle Mean (SD) (N)	High Mean (SD) (N)
Weight (kg)	6.48 (4.50) (27)	9.47 (4.58) (34)	11.36 (4.65) (19)
F weight	5.91 (4.41) (14)	9.83 (4.28) (12)	11.23 (2.09) (9)
M weight	7.1 (4.69) (13)	9.28 (4.82) (22)	11.47 (6.27) (10)
Nitrogen (δ^15^N)	6.32 (0.69) (46)	5.31 (0.60) (35)	5.65 (0.66) (66)
F Nitrogen	6.48 (0.75) (21)	5.47 (0.55) (19)	5.64 (0.91) (32)
M Nitrogen	6.15 (0.62) (18)	5.12 (0.66) (12)	5.60 (0.81) (32)
Carbon (δ^13^C)	− 21.89 (0.93) (46)	− 21.86 (0.42) (35)	− 21.75 (0.49) (66)
F Carbon	− 21.75 (0.97) (21)	-21.97 (0.49) (19)	− 21.82 (0.50) (32)
M Carbon	− 21.87 (0.94) (18)	− 21.73 (0.30) (12)	− 21.71 (0.48) (32)

### Data collection

Hair samples and weight measurements were collected between 2000 and 2006. GONHS regularly traps the macaques across the Upper Rock Nature Reserve to maintain a detailed data base with biometric information for each individual, including weight on a sling scale, as well as to microchip, test, and vaccinate the population. The data analyzed in this study were collected during these regular trappings, including the weight data, which were obtained from 80 individuals across six distinct macaque troops. Isotopic data were obtained through stable isotope analyses of hair samples at the University of Notre Dame. Hair was bundled into approximately 0.1 mg of total hair per mm of sample length, washed with acetone to remove contaminants, and then dried for analysis. Samples were enclosed in tin foil cups and combusted in a Carlo-Erba elemental analyzer for analysis using the continuous flow isotope ratio mass spectrometer (Finnigan Delta Plus). Carbon-13/Carbon-12 and Nitrogen-15/Nitrogen-14 ratios are reported as δ values in parts per thousand or per million^19^.

As noted, the field-based data collections and isotope analyses were conducted separately, and in the process of shipping and processing the samples for the isotope analyses the individuals were ascribed new IDs. The two sets of IDs became decoupled and thus, the two data sets cannot be directly linked. We also do not have age class data for the isotope data. We address this as a limitation in the Discussion.

### Ethics approval

The Gibraltar government, veterinary services of Gibraltar, and the University of Notre Dame Institutional Animal Care and Use Committee approved this research (protocol numbers 06-067 and 11-014). All data collection was performed in accordance with the expectations and guidelines of these institutions and committees. This study has been reported according to the ARRIVE guidelines.

### Statistical analyses

We ran all statistical analyses and created figures in R Studio (2023), version 4.3.1. The core dependent variables approximated normal distributions (i.e., δ^13^C, δ^15^N, body weight; Table 2). We first ran ordinary least squared (OLS) linear regression interaction models to test whether sex moderated the relationships between anthropogenic food exposure with δ^13^C values, δ^15^N values, and body weight, respectively. For the body weight model, we adjusted for age class as a covariate; age data were not available for δ^13^C and δ^15^N models. If the interaction terms were not statistically significant, we conducted complementary regression models focusing on the main effects (without the interaction terms) to test whether anthropogenic food exposure and sex predicted the dependent variables. We conducted the regression models in this order because any main effects of anthropogenic food exposure or sex would be conditional to the interaction term if the latter was statistically significant. We tested these models for heteroskedasticity using the Breusch-Pagan Test. We calculated standard deviation units for key results related to body weight to characterize the magnitude of the effect sizes. We evaluated statistical significance at p < 0.05. Figures were created in R Studio using ggeffects^35^ and sjPlot^36^ packages.

## Results

In OLS linear regression models for δ^13^C values, we did not observe statistically significant moderation effects (anthropogenic food exposure × sex; both p > 0.2) or main effects (all p > 0.5; Table 3).

**Table 3:** OLS regression model results comparing Gibraltar monkey isotopic Carbon values based on levels of exposure to anthropogenic foods with the Low category as the reference.

Interaction model (M & F; N = 147)				Main effects model (M & F; N = 147)		
	b	95% CI	p	b	95% CI	p
Category						
Middle	0.14	− 0.34, 0.62	0.567	− 0.06	− 0.37, 0.24	0.679
High	0.16	− 0.22, 0.54	0.413	0.04	− 0.22, 0.30	0.787
Female	0.12	− 0.29, 0.53	0.568	− 0.07	− 0.29, 0.15	0.533
Interaction						
Middle × Female	− 0.36	− 0.98, 0.27	0.265			
High × Female	− 0.23	− 0.75, 0.29	0.385			
R^2^		0.02			0.01	

**Figure 2:**
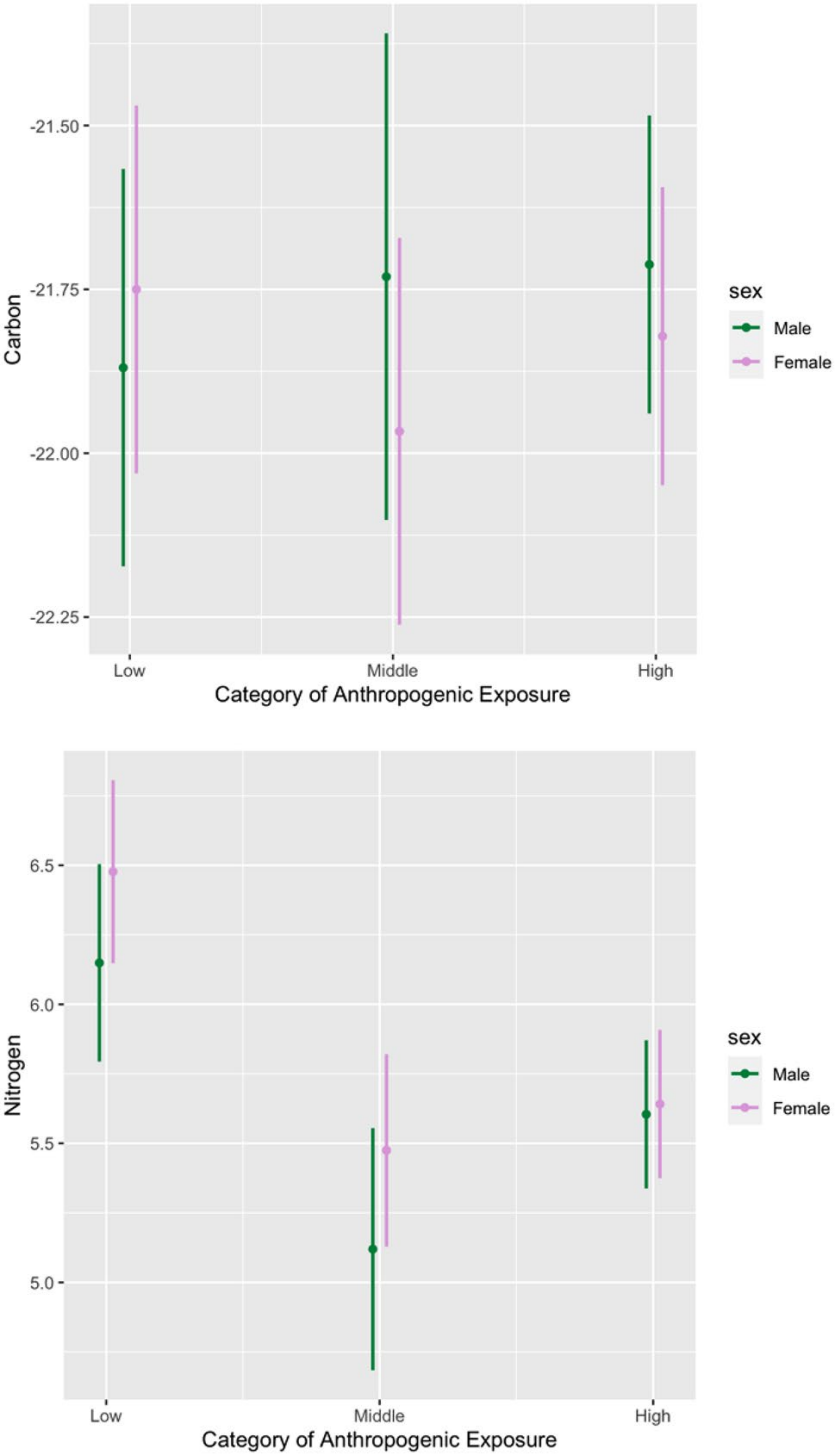
Plots of OLS regression models predicting Carbon-13 (top) and Nitrogen-15 (bottom) isotopic concentrations by category. C-13 values were not significantly predicted by category or sex, whereas N-15 values significantly differed by category. C-13 and N-15 values (y-axes) per category are centered at their mean ± 1 standard deviation.

In comparable models predicting δ^15^N values, we found no significant interaction effects (both p > 0.3; Table 4). However, there were main effects for anthropogenic food exposure (Fig. 2). Specifically, δ^15^N values were lower on average in the Middle (p < 0.001) and High (p = 0.017) categories compared to the Low category, but were lowest in the Middle category. Males and females did not significantly differ for δ^15^N values (p > 0.1).

**Table 4:** Results of OLS regression models comparing Gibraltar monkey isotopic Nitrogen values based on levels of exposure to anthropogenic foods with the Low category as the reference.

Interaction model (M & F; N = 147)				Main effects model (M & F; N = 147)		
	b	95% CI	p	b	95% CI	p
Category						
**Middle**	− **1.03**	− **1.59,** − **0.47**	** < 0.001**	− **1.00**	− **1.37,** − **0.64**	** < 0.001**
**High**	− **0.54**	− **0.99,** − **0.10**	**0.017**	− **0.70**	− **1.00,** − **0.39**	** < 0.001**
Female	0.33	− 0.16, 0.81	0.183	0.19	− 0.07, 0.45	0.146
Interaction						
Middle × Female	0.03	− 0.71, 0.76	0.942			
High × Female	− 0.29	− 0.90, 0.32	0.350			
R^2^		0.22			0.21	

Significant results are noted in bold.

We found a significant moderation effect for (anthropogenic food exposure × sex) in predicting body weights when comparing low and high categories (p = 0.034; Table 5).

**Table 5:** Results of OLS regression interaction model comparing Gibraltar monkey weights based on levels of exposure to anthropogenic foods with the Low category as the reference.

Interaction model (M & F; N = 80)			
	b	95% CI	p
Category			
Middle	1.11	− 0.58, 2.79	0.196
**High**	**2.25**	**0.23, 4.28**	**0.030**
Female	− 0.14	− 1.98, 1.70	0.881
**Subadult**	− **6.87**	− **8.31,** − **5.43**	< **0.001**
**Juvenile**	− **9.94**	− **11.40,** − **8.49**	** < 0.001**
Interaction			
Middle × Female	1.54	− 4.28, 1.05	0.240
**High **× **Female**	− **3.16**	− **6.07,** − **0.25**	**0.034**
R^2^		0.79	

Significant results are noted in bold.

**Figure 3:**
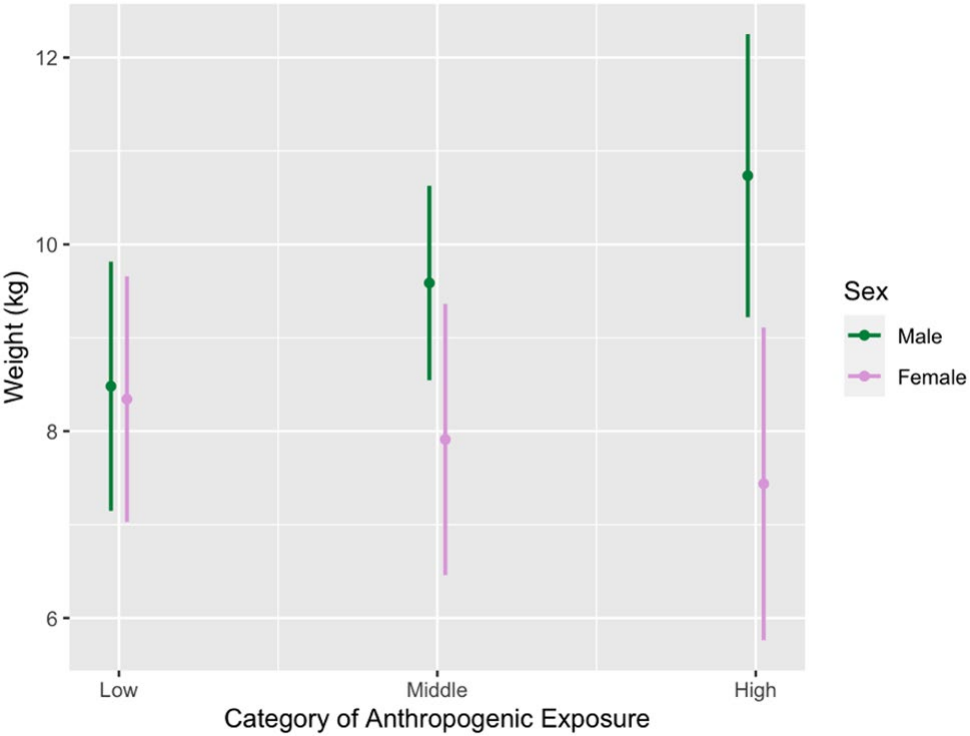
Plot of OLS regression model predicting weight by category, moderated by sex. An interaction effect is shown: as anthropogenic exposure increases, male body weights increase on average, whereas female body weights decrease on average. Weights (y-axis) for each category are centered at their mean ± 1 standard deviation.

As anthropogenic food exposure increased, male body weights were also higher, on average, while with greater anthropogenic food exposure female body weights were lower, on average. We show this interaction effect in Fig. 3, with the following effect sizes in standard deviation (SD) units for male–female differences by category: low (0.03), medium (0.34), and high (0.67).

## Discussion

In the present study, we built on prior work from this site^19^ to explore how differential exposure to anthropogenic foods is linked to variation in isotopic compositions and body weights. We found that δ^15^N values, but not δ^13^C values, significantly differed based on anthropogenic food exposure and that the links between anthropogenic food exposure and body weights were moderated by sex (Fig. 3). Our results are inconsistent with the previous study regarding δ^13^C values, which we discussed below, but nonetheless suggest a significant and meaningful association between anthropogenic food exposure and macaque troops’ feeding ecologies at URNR.

δ^15^N values differed based on exposure and access to anthropogenic foods. Lower δ^15^N values tend to reflect higher consumption of processed human foods^26^ whereas higher δ^15^N values are generally associated with the consumption of plants with greater nitrogen content through natural foraging^28^. We predicted δ^15^N values would be lower with increasing anthropogenic food exposure. Along these lines, the Middle and High categories had lower δ^15^N values compared to the Low category. However, the Middle category reflected the lowest δ^15^N values, overall. Troops comprising the Low category did not have access to anthropogenic foods and thus consumed a high portion of naturally occurring plants^19^, which likely explains their higher δ^15^N values, as expected. The High category includes the Incinerator troop, which is not provisioned with monkey chow by URNR. It is possible that these individuals slightly increased the High category’s δ^15^N values, as they forage for naturally occurring plants with high Nitrogen content to supplement the human foods available to them from the incinerator.

The previous study^19^ found that δ^13^C values of the Middle Hill troop, categorized in the current study as “Low”, significantly differed from the Ape’s Den and Prince Phillip’s Arch troops, categorized in the current study as “High”. In the current study, however, we found no significant or meaningful differences across any category for δ^13^C values, including between the Low and High categories. This discrepancy may be due to the addition of a troop in the “High” category that was not included in the previous study, the Incinerator troop.

Our study found no significant associations between sex with either δ^13^C or δ^15^N values. Although we must be cautious in over-interpreting null results, this pattern is consistent with the idea that both males and females were generally feeding on the same food types within exposure categories. However, our body weight results suggest that males and females within these categories may have differential access to quantities of these foods. These results align with our hypotheses based on prior literature that suggests that NHP males have priority access to highly desired foods^17,18^. In particular, male Barbary macaques in Gibraltar were observed interacting more frequently with humans, including in interactions related to food^3^, while adult male Barbary macaques in Algeria were observed consuming substantially more anthropogenic foods than female macaques due to both priority access to as well as more risky behavior^11^. In the previous study, Schurr et al. found that male macaques at URNR were in fact monopolizing human feeding interactions^19^. The present study shows the potential implications of these behavioral occurrences on body size—we suggest that males are likely consuming more anthropogenic foods than females within troops and are heavier because of it.

Studies of wild populations anecdotally report that NHP individuals that consume more anthropogenic foods typically see weight gain, but few studies have analyzed this^11^. The results of the present study are consistent with the idea that greater exposure to anthropogenic foods contributes to increased body size depending on sex-based access to these foods. This differential exposure can lead to heavier male, but not female, macaques, which may result in differential health profiles for females and males within troops.

As a result of data management and labeling discrepancies between field and laboratory processes, the weight and stable isotope data could not be linked across individuals. For this reason, we analyzed these data separately. This does not affect the overall interpretation of the category differences for either type of data. However, consistent individualized data would have allowed for more complex and complete analyses regarding the relationships amongst body weights, isotopic compositions, and anthropogenic foods. Age data were also unavailable for individuals from whom we had stable isotope data, and those analyses did not include age as a covariate for this reason. Additionally, data on dominance rank were unavailable for inclusion in this study. Just as males typically have more access to preferred foods than female macaques, dominance hierarchies and age shape preferred food access and consumption. Higher ranked individuals typically have higher access to these foods because of their status, and younger individuals are more likely to exhibit risky behavior and engage with tourists for these foods^19^. Associated individualized rank and age data thus would have also allowed for more complete analyses and understandings of how body weights, isotopic compositions, and anthropogenic foods relate to each other (e.g., within male and female hierarchies).

Building from complementary prior behavioral observation work from this site^19^, we showed how isotopic values and body weights can be used to better understand how anthropogenic foods can be incorporated into wild macaque diets and differences in body size. The current study suggests that some macaques with higher exposure to anthropogenic food sources do consume those foods more often than macaques with lower exposure to them, and that this relates to higher—potentially atypical—body weight, especially for male macaques. This differential impact of anthropogenic foods on body weight based on sex have possible health implications, based on past results from captive populations showing that NHPs exposed to processed human foods can develop phenotypes consistent with obesity and Type II diabetes^22,37^. Though studies show that male NHPs typically have higher access to and consume more human foods than females, much of the primate literature on health outcomes of anthropogenic diets focuses on female NHP bodies^23,38^. Moreover, these and related studies have been conducted on captive populations, whereas studies of free ranging populations measuring health related to anthropogenic food consumption are sparse. As non-captive, free ranging populations continue to rely on or prefer human foods, it is important to assess the long-term health and fitness effects from their consumption. Incorporating body weight analyses is a key first step, but longitudinal studies of free ranging NHP populations that include more expansive health assessments can allow for a clearer understanding of the evolutionary implications of anthropogenic food on health, survival, and fitness. As NHP populations across the globe increase their reliance on anthropogenic food sources to survive, it is important that we expand our understanding on how these foods impact their health.

### Supplementary Information

Supplementary Information 1.

Supplementary Information 2.

## Data Availability

All data used in analyses are uploaded and available as an Excel file in the Supplementary Material. The Excel file has three sheets: (1) article and coding information; (2) the isotope data by individual and; (3) the weight data by individual. These data allow for replication of the present analyses. A pdf file is also included with the RStudio script used for the main OLS regression analyses.
