# Supplementary material for: Barbary macaques show sex-related differences in body weight based on anthropogenic food exposure despite comparable female–male stable isotope ratios
Source: Sci Rep. 2024 Feb 9;14:3320. doi: 10.1038/s41598-024-53641-9 (PMC10858218; doi:10.1038/s41598-024-53641-9)
Supplement: Supplementary file 2 — Supplementary Information 2. [file 41598_2024_53641_MOESM2_ESM.pdf]

1. Script used in RStudio for ordinary least squares (OLS) regression interaction model for weight, adjusting for age class as a covariate.

```
#read data; Excel sheets must be saved as separate .csv files
weights <- read.csv("Weight Data.csv", header = T)
#set comparison levels (low, male, adult)
category <- factor(weights$Category, levels = c("Low","Middle", "High"))
sex <- factor(weights$Sex, levels = c("Male","Female"))
age <- factor(weights$Age, levels = c("adult","subadult", "juvenile"))

#interaction model
lm <- lm(Weight ~ category*sex + age, data = weights)
summary(lm)
confint(lm, level=0.95)
```

2. Script used in RStudio for ordinary least squares (OLS) regression interaction and main effects models for $\delta^{15}N$ values.

```
#read data; Excel sheets must be saved as separate .csv files
isotopes <- read.csv("Isotope Data.csv", header = T)
#set comparison levels (low, male, adult)
category <- factor(isotopes$Category, levels = c("Low","Middle", "High"))
sex <- factor(isotopes$Sex, levels = c("Male","Female"))

#interaction model
lmN <-lm(N ~ category*sex, data = isotopes)
summary(lmN)
confint(lmN, level=0.95)

#main effects model adjusting for sex as covariate
Main_N <-lm(N ~ category + sex, data = isotopes)
summary(Main_N)
confint(Main_N, level=0.95)
```

3. Script used in RStudio for ordinary least squares (OLS) regression interaction and main effects models for $\delta^{13}C$ values.

```
#read data; Excel sheets must be saved as separate .csv files
isotopes <- read.csv("Isotope Data.csv", header = T)
#set comparison levels (low, male, adult)
category <- factor(isotopes$Category, levels = c("Low","Middle", "High"))
sex <- factor(isotopes$Sex, levels = c("Male","Female"))

#interaction model
lmC <-lm(C ~ category*sex, data = isotopes)
summary(lmC)
```

```
confint(lmC, level=0.95)

#main effects model adjusting for sex as covariate
Main_C <-lm(C ~ category + sex, data = isotopes)
summary(Main_C)
confint(Main_C, level=0.95)
```
